# Initial assemblage characteristics determine the functional dynamics of flower‐strip plant communities

**DOI:** 10.1002/ece3.9435

**Published:** 2022-10-18

**Authors:** Antoine Gardarin, Muriel Valantin‐Morison

**Affiliations:** ^1^ UMR Agronomie, INRAE, AgroParisTech Université Paris‐Saclay Thiverval‐Grignon France

**Keywords:** community structure, functional diversity, functional redundancy, functional traits, seed mixture, species richness

## Abstract

In agroecosystems, species‐rich habitats, such as linear field margins and flower strips, are beneficial to the overall biodiversity and contribute to pest control. Their effects are thought to be mediated by plant species composition and diversity. However, the management of plant communities with targeted levels of functional diversity has been little investigated. In an open field landscape, we compared the effects of the sown species richness (9, 14, and 29 species) and functional diversity (high vs. low) of eight different seed mixtures, sown in flower strips, on the 4‐year temporal dynamics of their functional diversity. There was a good agreement between the expected and realized species richness and functional diversity at the start of the experiment. All plant assemblages progressively lost species over time, but this decline was lower for assemblages sown with a high initial functional diversity, in which species evenness was maintained at higher levels. Species‐rich assemblages had a higher degree of functional redundancy, and their functional diversity remained higher over time than less rich assemblages. A possible explanation for this is that functional redundancy would have enabled the compensation for the loss of species by functionally equivalent species. The realized functional diversity of the sown species also limited the establishment of spontaneous species, perhaps due to a higher degree of niche occupancy. This study provides useful insight into the creation of functionally diversified plant communities. A high level of initial species and functional diversity is required to guarantee a greater temporal persistence of the communities.

## Graphical abstract

We present the results of a 4‐year field experiment, during which we compared eight plant assemblages differing in species richness, functional diversity, and functional redundancy. We found that the initial levels of plant diversity and functional redundancy strongly explained the temporal dynamics of the functional diversity of the communities. This functional diversity remained higher throughout the experiment for the assemblages with higher initial species richness and functional diversities.

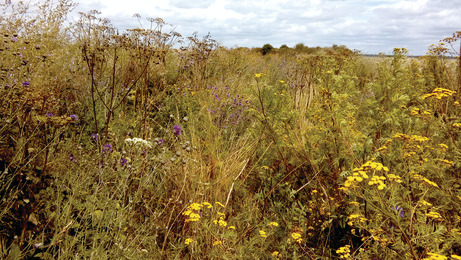

## 1 INTRODUCTION

Biodiversity plays a key role in the functioning of natural and managed ecosystems and in the services they deliver (van der Plas, 2019), such as primary production, herbivore regulation, pollination, and biogeochemical cycles. Numerous studies have demonstrated the positive role of species richness in the supply of ecosystem services and in ensuring the stability of this supply in the face of environmental disturbances and changes (Weisser et al., 2017). In particular, plant species richness can lead to changes in the functional composition of plant and animal communities (Edlinger et al., 2020).

**FIGURE 1:**
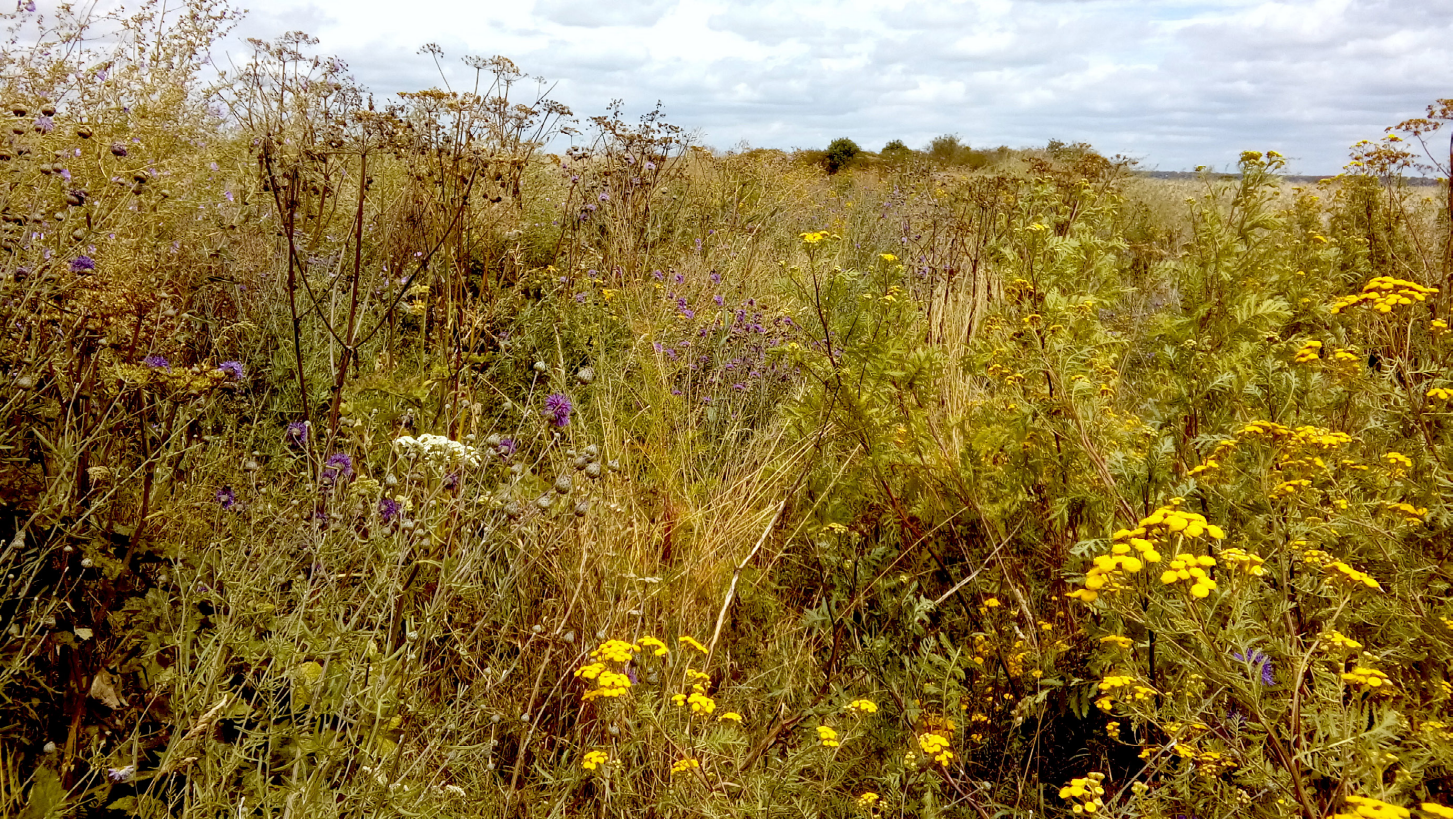
Perennial and diversified flower strips provide shelters and trophic resources for numerous components of the biodiversity in agroecosystems (photo credit: A. Gardarin).

In agroecosystems, species‐rich habitats, such as linear field margins and flower strips (Figure 1) between or within crop fields, act as refuges for a large range of plant species and are beneficial in terms of overall biodiversity (Holland et al., 2017). In particular, they support populations of pest predators, parasitoids, and pollinators, thereby contributing to conservation biological control by reducing pest abundance (Albrecht et al., 2020) and to entomophilous pollination. Their effects, particularly those on high trophic levels, are mediated by plant species composition and diversity (Gardarin et al., 2021; Haaland et al., 2011). Plant communities with high levels of taxonomic and functional diversity enhance diversity and multifunctionality across trophic levels (Lefcheck et al., 2015). High levels of functional diversity in plant communities should support more abundant and diversified animal communities through several mechanisms. This includes the supply of complementary habitats and resources required by animals at different stages of their life cycle (Wäckers et al., 2005), the supply of a diversified diet, resulting in greater animal fitness (Filipiak, 2019), the provision of more niches for consumers, offering more opportunities for the first trophic level (Junker et al., 2013), and the provision of more complex habitats, lowering the risk of intra‐guild predation (Griffiths et al., 2008).

Despite the importance of vegetation composition, little work has examined the factors that determine the achievement of objectives in terms of species composition when implementing flower strips, and even more so when aiming for high functional diversity (Ebeling et al., 2018; Laughlin et al., 2018). For example, in habitat creation or restoration projects, theoretical approaches have been developed to select lists of species to achieve specific objectives in terms of functional composition (Laughlin et al., 2017; M'Gonigle et al., 2017) and less frequently functional diversity (Giannini et al., 2017; Santala et al., 2022). The latter studies hypothesized that high functional diversity and species numbers would lead to resilient and invasion‐resistant communities. The assemblages obtained have however not been evaluated under real conditions. Very few have indeed investigated whether sowing a seed assemblage resulted in a plant species composition with the desired plant functional diversity aimed at enhancing pollinators or natural enemies (Balzan et al., 2014; Uyttenbroeck et al., 2015). Marshall et al. (1994) found, in a comparison of different seed assemblages, that habitats sown with large numbers of species resulted in a higher realized richness than habitats sown with fewer species. In addition, a steady decrease in the number of sown plant species has been recorded with succession in some studies (De Cauwer et al., 2005), potentially modifying the functional composition of plant communities. This modification could depend on the initial composition of the communities since some mixes with perennial species maintained very well their diversity, as observed in other studies (Ouvrard et al., 2018).

The dynamics of spontaneous or synthetic plant communities have been extensively studied from the point of view of plant–plant interactions, with functional approaches based on plant response traits involved in competitive interactions (Roscher et al., 2013). However, the consequences of these dynamics on the functional diversity of plant communities, examined from the point of view of interactions with arthropods, have been much less explored. Given these knowledge gaps, we took advantage of an experimental design, initially conceived to study the role of composition and functional diversity on arthropods and biological control, to explore whether the sown diversity affects the temporal dynamics of plant communities and the functional diversity of traits involved in plant‐insect interactions.

Firstly, we asked how the initial species richness and level of functional diversity of different plant assemblages affect the multi‐year dynamics of the realized functional diversity. The decrease of plant species richness through time, frequently observed in sown flower strips or agri‐environmental schemes (De Cauwer et al., 2005; Pfiffner & Wyss, 2004), should modify the functional diversity of plant communities. However, at a given level of sown functional diversity, greater species richness can be associated with greater functional redundancy, which can compensate for the temporal fluctuations in the abundance of some species (de Bello et al., 2021). Functional redundancy, occurring when species contribute similarly to ecosystem function, can enhance the temporal stability of functional diversity against the loss of individual species through compensation delivered by functionally equivalent species, as previously shown for ecosystem functions (Burkle et al., 2020; Hooper et al., 2005). We therefore expected that greater species richness would have a stabilizing effect on the temporal dynamics of the realized plant functional diversity.

Secondly, we wondered how competitive interactions between species could explain the temporal dynamics of the assemblages. During succession, species with a resource acquisition strategy are progressively excluded by competitive species that conserve the nutrients they acquire (Garnier et al., 2016). This results in an increase over time in species with a resource conservation strategy, that is, with a high leaf dry matter content, low specific leaf area, and with vegetative propagation, because these traits capture the resource acquisition and conservation strategies (Garnier et al., 2016). We therefore expected that, during succession, the dynamics of the functional composition of the different assemblages would be explained by regeneration strategies and competitive interactions within the communities.

Thirdly, we asked whether higher plant functional diversity confers better resistance to the spontaneous establishment of unsown species. Communities initially sown with a higher number of species are more resistant to spontaneous invasion of unsown species (Hector et al., 2001; Weisser et al., 2017). Given the assumed predominant role of competitive interspecific interactions in perennial plant communities, a greater functional diversity of traits would also increase niche occupancy, with fewer available resources for an invading species (Gallien & Carboni, 2017; Weisser et al., 2017). In our experiment, we therefore expected that the abundance of spontaneously arriving species should decrease with increasing sown species richness and sown functional diversity.

To summarize, our objectives were to assess (1) if the initial species richness and level of functional diversity of different plant assemblages affect the multi‐year dynamics of the realized plant functional diversity, (2) how far can regeneration and competition strategies between species explain the temporal dynamics in the different assemblages, and (3) whether functionally diversified communities are more resistant to invasions by spontaneously arriving species.

## 2 MATERIALS AND METHODS

The experiment was conducted between 2013 and 2017, in a 13 ha field at Grignon, France (N 48.837°, E 1.956°), with a degraded oceanic climate (Figure S1). The field had a deep loamy clay soil, with a decreasing gradient of soil depth from north to south. The experimental field was divided into three blocks, running in a north–south direction, to account for this soil heterogeneity. Apart from the presence of a small woodland (2500 m^2^) next to the northern side of the field, it was surrounded by a very simplified open field landscape cropped under conventional farming.

### 2.1 Design of species assemblages with contrasting species and functional diversities

Using literature data (Table S1), we built eight plant assemblages (Appendix S1) by varying species richness (low, medium, or high, named LS, MS, and HS, respectively), the functional diversity of traits (low or high, named LF and HF, respectively) and species identity (two sets of species). For designing the assemblages, we focused on a few traits involved in plant‐arthropod interactions and easily accessible in databases (Table 1): (1) provision of flower resources, that is, floral and extrafloral nectar or pollen, (2) temporal availability of the resource, that is, the duration of the flowering period, (3) accessibility of the resource, depending on flower shape, and (4) provision of habitats through height at flowering. We crossed orthogonally the species richness and functional diversity gradients in an unbalanced design. It was indeed not possible to combine every level of each factor due to space constraints. The range of species richness was chosen to encompass the range of observed species richness in field margins in France (Alignier, 2018; Fried et al., 2018). By combining these two factors, we obtained four categories of plant assemblages:

1. Low functional diversity and medium species richness (14 species), LFMS;
2. High functional diversity and low species richness (9 species), HFLS;
3. High functional diversity and medium species richness (14 species), HFMS;
4. High functional diversity and high species richness (29 species), HFHS.

**TABLE 1:** Relationships between the plant traits used in this study and their functional significance toward plant–arthropod interactions and toward plant–plant competitive interactions.

Category	Trait (or plant strategy)	Data type	Functional significance
**Plant‐arthropod interactions**			
Provision of trophic resources	Amount of floral nectar	Numerical	The carbohydrate and protein resources provided by plants increase the longevity, reproduction, and dispersal of their consumers (Wäckers et al., 2005).
	Provision of extrafloral nectar	Categorical	Extrafloral nectar is more easily accessible to arthropods with short mouthparts (Heimpel & Jervis, 2005). It generally has a higher sugar concentration than nectar (Lee et al., 2004).
Attractiveness of the trophic resource	Flower or inflorescence diameter	Numerical	Visual signals, such as plant height, flower height, inflorescence size, and color, are involved in resource detection, and a high degree of visual attractiveness increases the abundance of natural enemies (Fiedler & Landis, 2007; Hatt et al., 2018).
	Basic flower color	Categorical	
	UV reflectance pattern	Categorical	
Temporal availability of trophic resources	Week of onset of flowering	Numerical	The phenological match between flowering period and arthropod floral resource requirements is crucial for completion of the life cycles of flower‐visiting arthropods (Welch & Harwood, 2014).
	Duration of flowering	Numerical	
Accessibility of the trophic resource	Nectar depth	Numerical	A correlation between nectar holder depth and the proboscis length of the flower visitor has been observed in several insect groups, especially in pollinators. As a result, flower size is one of the most important variables determining the abundance and diversity of flower visitors (Heimpel & Jervis, 2005; van Rijn & Wäckers, 2016).
Provision of physical habitats	Leaf distribution	Categorical	Plant structural traits shape arthropod habitats (Parolin et al., 2012). Plants with a complex morphology have higher invertebrate abundances and biomasses than plants with a simple morphology (Hansen et al., 2010).
	Flowering height	Numerical	
	Raunkiær life form	Categorical	
**Plant–plant interactions**	Grime strategy (C and R scores)	Numerical	These different strategies describe the ability of established plants to grow and to reproduce along a resource and a disturbance gradients (Grime, 2001).
	Raunkiær life form	Categorical	This trait is related to the space occupation during seasons with adverse conditions.
	Leaf distribution	Categorical	These traits captures plants' light interception and the response to shading (Colbach et al., 2019).
	Week of onset of flowering	Numerical	Plant reproductive phenology is related to the temporal dynamics of plant biomass. A diversity of phenologies may enable the exploitation of different temporal niches and enable the coexistence of a larger number of species.
	Duration of flowering	Numerical	
	Flowering height	Numerical	Plant height represents a surrogate of the ability to compete for light with neighboring plants.
	Specific leaf area	Numerical	These traits are strong markers of the strategy of resource use and can be used to estimate the position of a species along the continuum between the acquisition and conservation of resources (Weiher et al., 1999).
	Leaf dry matter content	Numerical	

For each of these four categories, we designed two assemblages with different species identities, belonging to two distinct species lists, resulting in eight plant assemblages (Table 2). This allowed us to ensure independence between the factors tested and the identity of the species composing the assemblages.

**TABLE 2:** Composition of the eight assemblages differing in functional dispersion and species richness

	LFMS1	LFMS2	HFLS1	HFLS2	HFMS1	HFMS2	HFHS1	HFHS2
Sown functional dispersion	Low	Low	High	High	High	High	High	High
Sown species richness	Medium	Medium	Low	Low	Medium	Medium	High	High
Functional redundancy	0.890	0.889	0.836	0.836	0.871	0.875	0.918	0.913
Sown functional dispersion	0.191	0.202	0.220	0.218	0.248	0.229	0.245	0.230
Dicotyledonous species	11	11	6	6	11	11	26	26
Poaceae species	3	3	3	3	3	3	3	3
Number of sown species	14	14	9	9	14	14	29	29
Dicotyledonous species								
*Anthriscus sylvestris* (L.) Hoffm.	x						x	
*Arctium minus* (Hill) Bernh.	x						x	
*Foeniculum vulgare* Mill.	x						x	
*Hesperis matronalis* L.	x						x	
*Leucanthemum vulgare* Lam.	x						x	
*Medicago sativa* L.	x						x	
*Coronilla varia* L.	x						x	
*Trifolium pratense* L.	x						x	
*Achillea millefolium* L.	x		x		x		x	
*Alliaria petiolata* Cavara & Grande	x		x		x		x	
*Heracleum sphondylium* L.	x		x		x		x	
*Cyanus segetum* L.			x		x		x	
*Trifolium repens* L.			x		x		x	
*Veronica hederifolia* L.			x		x		x	
*Centaurea scabiosa* L.					x		x	
*Euphorbia cyparissias* L.					x		x	
*Hypericum perforatum* L.					x		x	
*Tanacetum vulgare* L.					x		x	
*Verbascum densiflorum* Bertol.					x		x	
*Ajuga reptans* L.							x	
*Bellis perennis* L.							x	
*Capsella bursa‐pastoris* (L.) Med.							x	
*Echium vulgare* L.							x	
*Galium odoratum* (L.) Scop.							x	
*Malva sylvestris* L.							x	
*Potentilla reptans* L.							x	
*Carum carvi* L.		x						x
*Cynoglossum officinale* L.		x						x
*Daucus carota* L.		x						x
*Hypochaeris radicata* L.		x						x
*Jacobaea vulgaris* L.		x						x
*Lotus corniculatus* L.		x						x
*Onobrychis viciifolia* Scop.		x						x
*Melilotus altissimus* Thuill.		x						x
*Barbarea vulgaris* R. Br.		x		x		x		x
*Cota tinctoria* (L.) J.Gay ex Guss.		x		x		x		x
*Pastinaca sativa* L.		x		x		x		x
*Medicago lupulina* L.				x		x		x
*Stellaria media* (L.) Vill.				x		x		x
*Vicia sativa* L.				x		x		x
*Cichorium intybus* L.						x		x
*Galium mollugo* L.						x		x
*Knautia arvensis* (L.) Coult.						x		x
*Plantago lanceolata* L.						x		x
*Verbascum lychnitis* L.						x		x
*Geum urbanum* L.								x
*Glechoma hederacea* L.								x
*Lamium album* L.								x
*Ranunculus repens* L.								x
*Reseda luteola* L.								x
*Taraxacum sect. Ruderalia* Wiggers								x
*Veronica persica* Poir.								x
Poaceae								
*Arrhenatherum elatius* (L.) P.Beauv.	x	x	x	x	x	x	x	x
*Dactylis glomerata* L.	x	x	x	x	x	x	x	x
*Schedonorus arundinaceus* Schreb.	x	x	x	x	x	x	x	x

*Note*: The assemblages had a low or high functional dispersion (LF or HF), a low, medium, or high species richness (LS, MS, or HS). They were composed of dicotyledonous species from two different lists (1 or 2) completed with the same three grass species. Plants were named according to French taxonomic referential (TaxRef v12; Gargominy et al., 2018).

Although this approach for designing plant assemblages was arbitrary, we verified that they were consistently ranked across a functional diversity gradient.

Functional diversity is a conceptual variable and was computed through the functional dispersion (FDis), metrics, which reflects niche diversity. Functional dispersion is the abundance‐weighted mean distance (Gower distance) of individual species from the centroid of all species in the trait space (Laliberté & Legendre, 2010). Functional dispersion was significantly lowest for the LF assemblages and highest for the HF assemblages with medium and high species richness (*p* = .021).

Across the assemblages with high functional diversity, the increase of species richness was expected to increase functional redundancy. To check for this, the functional redundancy of the sown assemblages was calculated as the difference between the Simpson index and Rao's quadratic diversity (de Bello et al., 2007; Pillar et al., 2013). Functional redundancy differed significantly between each of the four types of assemblages (*p* < 10^−4^). Thus, at high functional dispersion, an increase in initial species richness effectively resulted in an increase in functional redundancy.

Plant species (Table 2) were chosen among those typically found in field edges in the region. Seed assemblages (Table S2) were constituted with commercial seeds, with ecotypes of local origin (northern part of the Parisian Basin, France) where possible. Within each assemblage, the species were present in the same proportions in terms of seed numbers, based on the measured thousand‐seed weight of each batch of seed.

### 2.2 Experimental design

Each assemblage was sown on a 6 × 44 m^2^ experimental plot, with six replicates, as shown in Figure S2, with each assemblage represented twice in each block. A control treatment, sown with the same crop species as the rest of the field, was also included in the experimental design. This treatment was disturbed each year for sowing the crop and was therefore not analyzed in this study which focused on the dynamics of the flower strips.

Before sowing, the soil was plowed and tilled with a rotary harrow. Seed assemblages were sown manually, at a density of 240 seeds·m^−2^ on October 15, 2013. A roller was then passed over the soil, to ensure good seed‐soil contact. In subsequent years, the vegetation was managed by means of a single cut, with removal of the residues, in November.

From the autumn of 2013 to the 2017 harvest, the crop succession was winter barley – maize – faba bean – oilseed rape in the west of lines 1–3, and maize – pea+barley intercrop – oilseed rape – winter wheat in the west of lines 4–6. Crops were managed with a mean total of three fungicide and five herbicide treatments over the 4 years, averaged in each field. We were very careful to ensure that there was no herbicide drift in the strips.

### 2.3 Vegetation analysis and functional characterization of the communities

Vegetation analysis was conducted once annually, in June, between 2014 and 2017. In each treatment, the vegetation was assessed in 3 × 15 m^2^ permanent plots located in a representative position within each experimental plot, at 1.5 m from each edge to avoid the interface zone between the crop and the strip. The percentage of the ground covered by each sown and spontaneously growing plant species was estimated by eye, by the same observer in each case. As there was overlap between species cover, the total cover could be higher than 100%. In 2015, only the three replicate lines numbered 4–6 (Figure S2) were assessed. As surveys were carried out in June, the cover of vernal species (some of them being small and senescent in June) could have been underestimated.

Trait values were either measured or retrieved from published data or online databases for the 151 observed species. In order to assess the ability of plant assemblages to support higher trophic levels (first objective) and to analyze the temporal dynamics of plant assemblages (second and third objectives), the traits analyzed (Table 1) were related to (1) plant–arthropod interactions (provision of trophic resources, temporal availability, accessibility, and attractiveness of the resource, provision of physical habitats; Gardarin et al., 2018), and (2) plant–plant interactions (plant morphology, resource acquisition, or conservation strategy). We checked that these traits were not highly correlated (correlation coefficient <0.70).

#### 2.3.1 Flower traits

Flower traits were measured, by a single observer, on plants found in the experimental field, or on plants found in the surrounding area, for the sown species that did not emerge (for comparison with the sown and the realized functional assemblage characteristics). Between 2015 and 2017, we recorded the species that were flowering (i.e., flowers present in at least 20% of the individuals). We determined two traits: the calendar week (numbered from 1 to 52) in which flowering began and the duration of flowering (numbers of weeks). We averaged the values of these traits over the 3 years of field observations. The presence of extrafloral nectar (0 = none, 1 = present) was also noted.

Flower traits were measured during peak flowering, on five or 10 flowers collected from different plants of spontaneously growing (unsown) and sown species, respectively. Flowers were sampled early in the morning and placed in water for at least 1 h before observation. We measured the diameter of the flower or inflorescence (for grouped flowers) under a binocular microscope or with a ruler (for flowers with a diameter >20 mm). Flowers were dissected under a binocular microscope (Leica M80, 60×) linked to a video camera (Moticam 10, Motic) and then measured with ImageJ v1.50i digital image analysis software. The amount of floral nectar per individual flower was visually approximated on a three‐point scale (0 = none, 1 = some, 2 = lots). We measured nectar depth as the distance between the uppermost part of the perianth and the uppermost part of the zone in which nectar accumulated.

These measurements were completed with published trait measurements. Data concerning basic flower color and the presence of a UV reflection pattern were extracted from the BiolFlor database (Kühn et al., 2004), and missing data were completed on the basis of personal observations (for color) and with data from the Floral Reflectance Database (Arnold et al., 2010).

#### 2.3.2 Plant vegetative and architectural traits

We used data for leaf distribution (rosette, semi‐rosette, or leaves distributed along the stem), flowering height, specific leaf area, and leaf dry matter content from the LEDA database (Kleyer et al., 2008), for Raunkiær life form (Julve, 1998) and for the Competitive and Ruderal scores (percentage) of Grime strategy (Bàrberi et al., 2018; Kühn et al., 2004; Pierce et al., 2017).

For each trait, values were obtained for more than 95% of the species, with the exception of nectar depth, for which data were available for only 81% of the species, but accounting for 99.6% of total plant cover. On average, trait data were available for 97% of the 151 species we observed, accounting for 99.9% of the total plant cover.

To deal with the first objective (temporal dynamics of functional diversity of plant traits involved in interactions with higher trophic levels), functional diversity was calculated on the basis of the 11 following traits: amount of floral nectar, presence of extrafloral nectar, flower or inflorescence diameter, flower color, presence of a UV reflection pattern, date of flowering onset, duration of flowering, nectar depth, flowering height, and leaf distribution. These traits were very weakly correlated (Figure S4), with few significant correlations and low correlation coefficients. Concerning the third objective (interactions within plant communities), we used the following traits: reproductive height, leaf distribution, Raunkiær life form, Grime strategy (C and R scores), date of flowering onset, duration of flowering (measurements of plant phenology), specific leaf area, and leaf dry matter content. There were more significant correlations between these traits involved in plant–plant interactions (especially between flowering height, Grime strategy, life form, and leaf distribution), but correlation coefficients did not exceed 0.70 between different traits (Figure S5). We concluded that these traits were not fully redundant.

### 2.4 Statistical analyses

#### 2.4.1 General model selection method

Statistical analyses were performed with R software, version 3.1.3 (R Core Team, 2020). We performed linear mixed models for continuous response variables (with Gaussian error distribution) and generalized linear mixed models (with Poisson error distribution) for discrete response variables. The models included plot number (numbered from 1 to 9 along the north–south transects, Figure S2) nested within transect number (from 1 to 6) as a random effect. Using a multimodel inference procedure (Burnham & Anderson, 2002), we fitted models by maximum likelihood methods, including all possible combinations of the predictors (fixed effects) and their interactions with *time since sowing* (fixed effect, continuous variable). We ranked all model candidates according to the Akaike Information Criterion (AIC). Models with a ΔAIC < 4 were selected (i.e., the difference to the lowest AIC of all models), and we present the statistical results for the full averaged model. If a single model was identified with a ΔAIC < 4, an analysis of variance and post hoc comparison tests (if relevant) were performed on this best model. In single models, the significance of the fixed effects was evaluated by type II analyses of deviance with Wald chi‐squared tests (Anova function from the car package). Pseudo‐*R* ^2^ was calculated with the function *r.squaredGLMM*. Analyses were carried out with the vegan (Oksanen, 2013), FD (Laliberté et al., 2014), lme4 (Bates et al., 2015), MuMIn (Bartoń, 2016), car (Fox & Weisberg, 2019), and emmeans (Lenth et al., 2019) packages. The distribution of model residuals (normality, homoscedasticity, outliers) was checked and confirmed with the *DHARMa* package (Hartig, 2019).

#### 2.4.2 Effect of the initial assemblage characteristics on the multi‐year dynamics of functional diversity

Three response variables were studied: realized functional dispersion, species richness, and Simpson's evenness of plant communities over the course of the experiment. The model selection approach was used to relate these variables to the initial assemblage characteristics: initial species richness (low, medium, high), initial functional diversity (low vs. high), and time (years since sowing, continuous variable).

To calculate functional dispersion of the assemblages, numerical traits with left‐skewed distributions (reproductive height, duration of flowering, flower size, and nectar depth) were log‐transformed before computing functional diversity metrics (Májeková et al., 2016). To verify that we used uncorrelated predictors, a principal components analysis was performed on several metrics of taxonomic and functional diversity commonly used in the literature (Figure S3), using the ade4 package (Dray & Dufour, 2007). The three‐species diversity and equitability indices (Shannon, Simpson, and Pielou) were highly related (Figure S3) and we kept Simpson's species evenness.

A weak point of our experimental scheme is that low initial functional dispersion was represented by two plant assemblages (with medium species richness), while high initial functional dispersion was represented by six plant assemblages (with low, medium, and high species richness). The realized species richness per time and functional dispersion in the observed plant communities were also related to each other. To better disentangle species richness from functional dispersion effects, two precautions were taken. First, we repeated all the statistical analyses on two subsets of assemblages: (1) we compared low and high functional dispersion while controlling for species richness (only medium species richness assemblages), and (2) we assessed the role of species richness across plant assemblages with high functional dispersion. Second, to estimate functional dispersion independently of species richness, we generated null functional dispersion values using the name‐shuffling approach (Swenson, 2014). Null models were realized by shuffling species names in the trait matrix. This approach maintains the observed patterns of trait co‐variance and of community structure and calculated the expected distribution of functional dispersion values given the observed species richness in the assemblage. This process was applied 1000 times to each observed plant assemblage. Then, standardized effect sizes (SES) were calculated for each observed plant assemblage as the observed functional dispersion minus the mean functional dispersion of the null distributions, and this value was divided by the standard deviation of the null distribution (Swenson, 2014). As above, a model selection procedure was used to relate the standardized functional dispersion to the initial assemblage characteristics. As the temporal variation of the standardized functional dispersion was not linear, the continuous variable *age* was replaced by a *year* categorical variable.

#### 2.4.3 Effect of regeneration and competition strategies on the dynamics of the assemblages

We first analyzed the temporal dynamics of the regeneration strategies through the use of a categorical composite trait combining lifespan and the type of regeneration (Bàrberi et al., 2018). The proportion of the ground covered by the species from each regeneration strategy category (annual, biennial, stationary perennial, aboveground creeping perennial, or belowground creeping perennial) was calculated and compared between years. For each regeneration strategy, we ran linear mixed models including year as a fixed effect and plot number nested within transect number as a random effect, followed by post hoc Tukey tests. We then analyzed the dynamics of competitive species across assemblages, by calculating the community‐weighted mean (CWM) of leaf dry matter content (LDMC) and specific leaf area (SLA) as these traits capture the resource acquisition and conservation strategies (Garnier et al., 2016). The CWM expresses the mean trait value weighted according to the relative abundances of each species at community level. Using linear mixed models, we analyzed the relationships between the CWM of LDMC and SLA on the one hand, and the initial assemblage characteristics on the other hand: initial species richness, initial functional dispersion (both categorical variables), and time (continuous variable).

#### 2.4.4 Effect of functional diversity on resistance to invasions

We investigated the effects of the sown species on the spontaneous vegetation on two response variables: total plant cover and species richness. As our objective here was to understand the interactions occurring within plant communities, we calculated functional dispersion of sown species with the nine traits involved in competitive interactions (Table 1). We first investigated the relationships between the two response variables for spontaneous vegetation (plant cover and richness) and the characteristics of the initial assemblage: initial species richness, initial functional dispersion (both categorical variables), and time since sowing (continuous). We then took into account the proportions of the sown species actually established, by calculating species richness and functional dispersion for the sown species only, from observations made during the 4 years of botanic assessments. In this second step, the explanatory variables were the observed richness, the observed functional dispersion of the sown species, and time since sowing.

## 3 RESULTS

### 3.1 How do the initial species richness and level of functional dispersion of different plant assemblages affect the multi‐year dynamics of realized functional dispersion of all traits?

**FIGURE 2:**
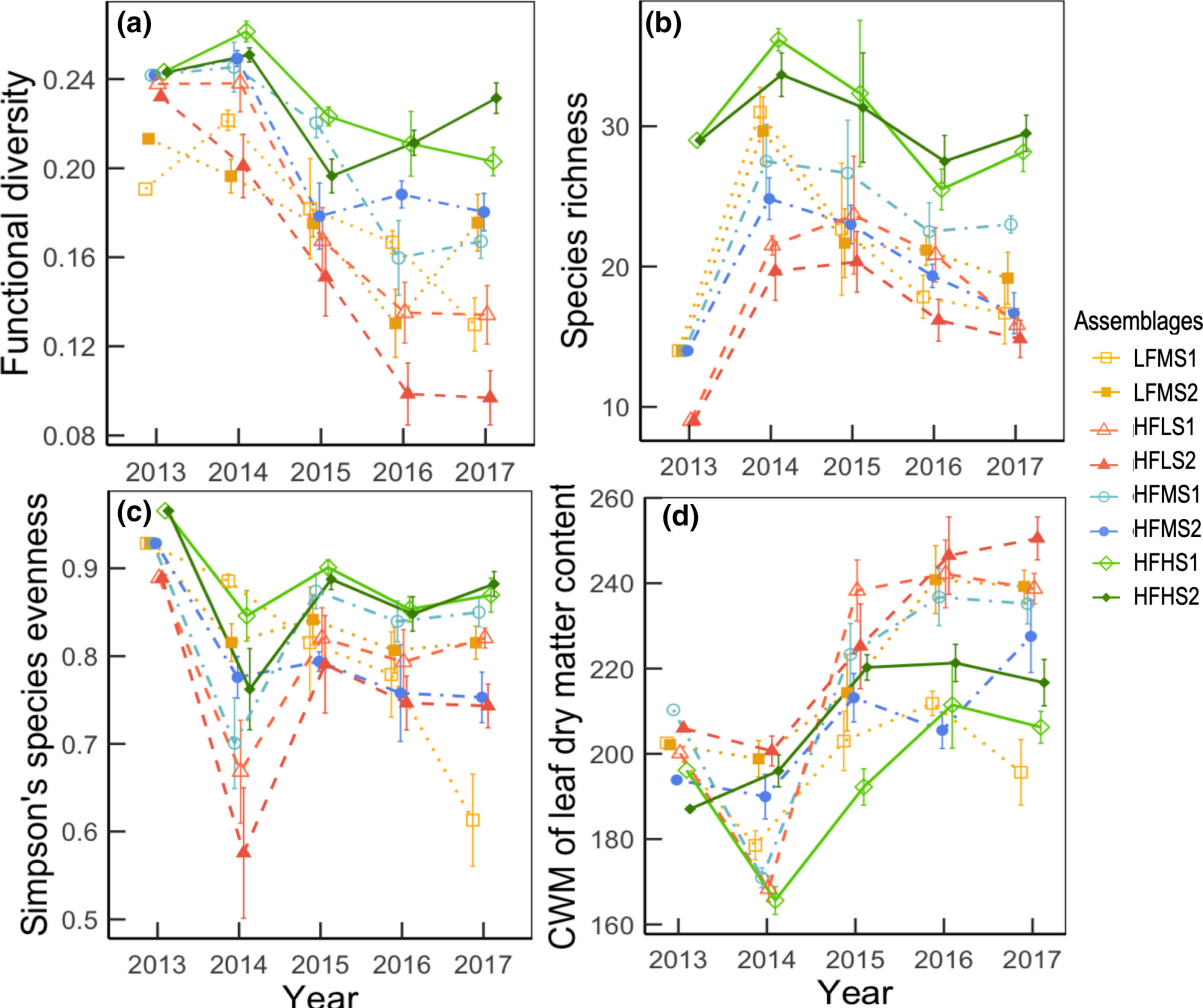
Dynamics of the taxonomic and functional diversity of the eight plant assemblages (means ± standard errors). The data for 2013 are based on the seed assemblages sown, and the data for the next 4 years were obtained from field observations, with six replicates per assemblage. The eight plant assemblages have a low or high functional dispersion (LF or HF), a low, medium, or high species richness (LS, MS, or HS) and are composed of species from two different lists (Table S2). CWM, community weighted mean.

The model best explaining the dynamics of functional dispersion of all traits, over the entire study period, selected with the multimodel inference procedure (Table 3), included a negative effect of time since sowing, a positive effect of initial functional dispersion and a positive “time × initial species richness” interaction. In general, the assemblages with high and low initial functional diversities (HF vs. LF) remained different (Table 3). Functional dispersion declined, at a rate dependent on initial sown species richness (Figure 2a, Table 3). The decrease in functional dispersion was greatest for the assemblages with the lowest initial species richness, intermediate for those with intermediate richness, and smallest for those with the highest species richness (Figure 2a). Consistent results were obtained with the standardized functional dispersion (Table S4), where there was also a positive effect of initial species richness. A high species richness therefore guaranteed the maintenance of a high level of functional dispersion.

**TABLE 3:** The initial richness and functional dispersion of plant assemblages affect their temporal dynamics

Response variable: functional dispersion of the whole plant community					
Explanatory fixed variables	Factor levels	Effect	*z* value	CI lower limit	CI upper limit
Intercept		0.213	11.217	0.175	0.250
Time		**−0.034**	5.788	**−0.046**	**−0.023**
Initial functional dispersion (ref = low)	High	**0.032**	2.253	**0.004**	**0.061**
Initial species richness (ref = low)	Medium	0.015	0.894	−0.018	0.048
	High	0.013	0.760	−0.021	0.048
Initial sp. richness × time (ref = low)	Medium × time	**0.012**	2.278	**0.002**	**0.023**
	High × time	**0.023**	4.063	**0.012**	**0.035**
Full model: marginal *R* ^2^ = .24; conditional *R* ^2^ = .25					

Response variable: species richness of the whole plant community					
Explanatory fixed variables	Factor levels	Effect	*z* value	CI lower limit	CI upper limit
Intercept		3.356	30.626	3.140	3.570
Time		**−0.181**	5.688	**−0.244**	**−0.119**
Initial species richness (ref = low)	Medium	**0.196**	2.580	**0.047**	**0.345**
	High	**0.481**	6.410	**0.334**	**0.627**
Initial functional dispersion (ref = low)	High	**−0.217**	2.146	**−0.415**	**−0.019**
Initial funct. dispersion × time (ref = low)	High × time	**0.093**	2.798	**0.028**	**0.158**
Initial sp. richness × time (ref = low)	Medium × time	0.000	0.027	−0.083	0.078
	High × time	0.003	0.170	−0.057	0.095
Full model: marginal *R* ^2^ = .37; conditional *R* ^2^ = .37					

Response variable: Simpson's species evenness of the whole plant community^a^					
Explanatory fixed variables	Factor levels	Effect	*z* value	CI lower limit	CI upper limit
Intercept		0.877	22.563	0.800	0.953
Time		**−0.042**	3.576	**−0.067**	**−0.020**
Initial species richness (ref = low)	Medium	0.028	1.557	−0.019	0.075
	High	**0.091**	3.804	**0.044**	**0.137**
Initial functional dispersion (ref = low)	High	**−0.168**	4.429	**−0.242**	**−0.095**
Initial funct. disp. × time (ref = low)	High × time	**0.064**	4.934	**0.038**	**0.089**
Initial sp. richness × time (ref = low)	Medium × time	−0.000	0.075	−0.034	0.028
	High × time	0.000	0.039	−0.032	0.029
Full model: marginal *R* ^2^ = .24; conditional *R* ^2^ = .25					

Response variable: community‐weighted mean of LDMC for the whole plant community					
Explanatory fixed variables	Factor levels	Effect	*z* value	CI lower limit	CI upper limit
Intercept		182.74	20.188	161.626	198.106
Time		**16.643**	5.166	**11.041**	**24.449**
Initial species richness (ref = low)	Medium	−0.869	0.839	−15.605	15.942
	High	−13.00	0.938	−17.563	15.146
Initial sp. richness × time (ref = low)	Medium × time	**−6.604**	2.382	**−11.116**	**−0.096**
	High × time	**−9.485**	2.963	**−14.118**	**−3.027**
Initial functional dispersion (ref = low)	High	−2.895	0.409	−23.601	12.114
Initial funct. disp. × time (ref = low)	High × time	1.531	0.556	−0.634	−10.371
Full model: marginal *R* ^2^ = .53; conditional *R* ^2^ = .59					

*Note*: In a 4‐year field experiment, we compared the functional dispersion, species richness, species evenness, and community‐weighted mean of leaf dry matter content of eight contrasting plant communities. These models are the full averaged models obtained after multimodel inference. Effects whose 95%‐confidence intervals (CI) do not encompass zero are in bold.

^a^ The three lowest values were outliers and were removed to reach normality.

In the fourth year of the experiment, about two‐thirds of the sown species were still present (66%, 68%, 71%, and 61% in the LFMS, HFLS, HFMS, and HFHS assemblages, respectively, Figure 2b). The observed species richness in the communities was explained by time since sowing (negative effect), initial species richness (positive effect), initial functional dispersion alone (negative effect), and in a positive interaction with time (Table 3). The number of species sown had a major effect (high slopes, Table 3).

As all species were sown at equivalent rates, in terms of seed numbers, Simpson's species evenness was very high in each of the assemblages sown in 2013, but differences appeared thereafter (Figure 2c). In general, evenness was lowest in LS and MS (Table 3). Like species richness, evenness decreased over time. Initial functional dispersion had a slight negative effect, but evenness decreased less over time in assemblages sown with a high versus low initial functional diversity, due to a positive interaction between functional dispersion and time (Table 3).

These results remained valid when we assessed (1) the effects of initial functional dispersion while maintaining for initial species richness constant in the subset of LFMS and HFMS assemblages, and (2) the effects of initial species richness across plant assemblages with a similar initial functional dispersion in the subset of HFLS, HFMS and HFHS assemblages (Table S3). The main difference was that the effect of initial functional dispersion on the realized species richness became not significant although this variable still interacted with time since sowing: the temporal decrease of species richness was significantly reduced in the assemblages with a high initial functional dispersion.

### 3.2 Can regeneration and competition strategies explain the dynamics in the different assemblages?

**FIGURE 3:**
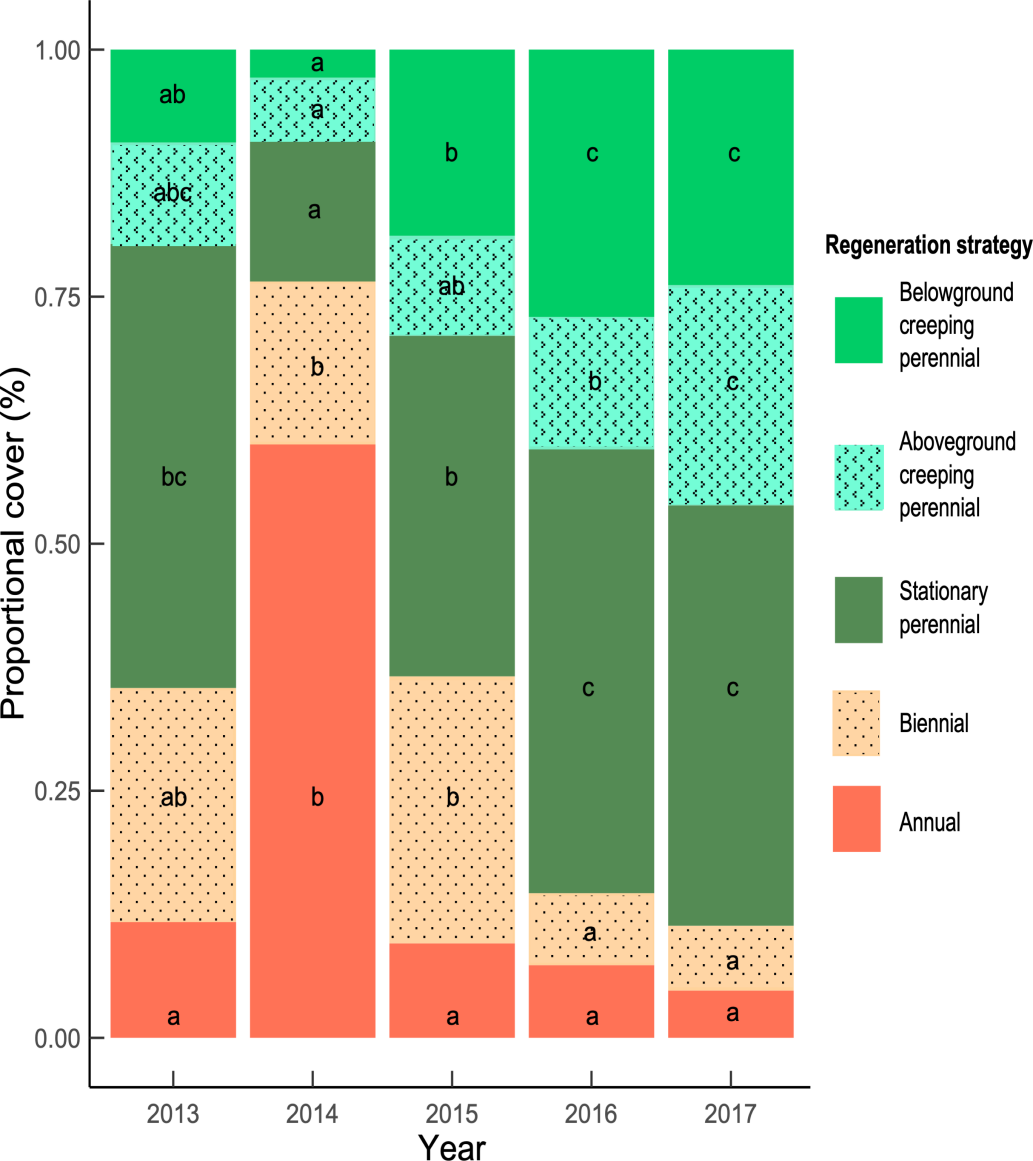
Temporal dynamics of the proportions of species, over all the tested assemblages, as a function of regeneration strategy. The data for 2013 are based on the seed assemblages sown, and the data for the next 4 years were obtained from field observations. The different letters indicate significant differences between groups (*p* < .05) and between years, within each category of regeneration strategy.

Averaged over all assemblages, plant cover for the five regeneration strategies varied considerably over the course of the experiment (Figure 3). In 2014, the first year after sowing, 60% of the total plant cover was occupied by annual sown and unsown species. The proportion of annual species decreased considerably thereafter. Biennial species accounted for about 20% of plant cover in 2014 and 2015, consistent with the proportion of biennial species sown, but species with this regeneration strategy declined after 2016. In parallel, the proportion of perennial species increased from 25% in 2014 to almost 90% in 2017. The proportions of stationary and belowground creeping perennials stabilized between 2016 and 2017, whereas the proportion of ground cover accounted for by aboveground creeping perennials continued to increase in 2017 (Figure 3).

During this 4‐year succession, the dynamics of the CWM of LDMC suggested a possible shift in community functional composition toward competitive species adapted to stable environments. The CWM of LDMC for the sown species assemblages was initially between 185 and 220 mg·g^−1^, depending on the assemblage considered; it then increased during the course of the 4‐year experiment (Figure 2d). This increase was significantly dependent on the initial level of species richness: the increase in LDMC was moderate in HS assemblages and largest in LS assemblages (Table 3). These results remained valid when we assessed separately the effects of initial functional dispersion or initial species richness while maintaining constant the other variable (Table S3).

On the contrary, there was no clear trend in the temporal dynamics of the CWM of SLA (Table S5) and there was no effect of any predictor.

### 3.3 Are functionally diversified communities more resistant to invasions by spontaneously arriving species?

We recorded 94 spontaneous species in the established plant communities, accounting for 17.3% of the total plant cover, on average, over the 4 years of the experiment. The spontaneous vegetation was dominated by *Helminthotheca echioides* L. (mean total cover of 2.1%), *Picris hieracioides* L. (1.3%), *Lysimachia arvensis* L. (1.1%), *Poa trivialis* L. (1.0%), *Papaver rhoeas* L. (0.9%), *Fallopia convolvulus* (L.) A. Löve (0.8%), *Cirsium arvense* (L.) Scop. (0.6%), and *Sonchus asper* (L.) Hill. (0.6%).

None of the initial characteristics (theoretical, at sowing) of the sown plant assemblages, in terms of initial species richness and initial functional dispersion, was related to the characteristics of the spontaneous vegetation, in terms of plant cover and species richness. For each of these two response variables, multimodel inference procedures systematically selected a single best model in which the sole response variable was *time* (results not shown). In each case, the percent cover and species richness of unsown plants decreased over time. Conversely, the functional dispersion of the observed sown species was negatively related to the richness of the spontaneous vegetation in the multivariate regressions (Table 4). The species richness of the spontaneous vegetation decreased with a higher observed functional dispersion of sown species. Concerning the total cover of spontaneous vegetation, there was no effect of functional dispersion of sown species (Table 4).

**TABLE 4:** The richness and functional dispersion of the *sown species* affect the richness but not the percent cover of the *spontaneous vegetation*.

Response variable: spontaneous vegetation cover				
Explanatory fixed variables	Effect	*z* value	CI lower limit	CI upper limit
Intercept	27.572	2.885	8.841	46.302
Time	−3.707	1.133	−10.124	2.708
Realized richness of sown species	0.505	1.121	−0.378	1.387
Realized rich. of sown sp. × time	−0.148	0.624	−0.613	−0.317
Realized funct. disp. of sown species	34.687	0.537	−91.902	161.276
Realized funct. disp. of sown sp. × time	−40.593	1.776	−85.390	4.204
Full model: marginal *R* ^2^ = .21; conditional *R* ^2^ = .45				

Response variable: species richness of spontaneous vegetation				
Explanatory fixed variables	Effect	*z* value	CI lower limit	CI upper limit
Intercept	3.547	10.953	2.912	4.182
Time	**−0.218**	2.268	**−0.407**	**−0.030**
Realized richness of sown species	0.001	0.070	−0.027	0.030
Realized rich. of sown sp. × time	0.008	1.834	−0.0005	0.0170
Realized funct. disp. of sown species	**−0.842**	2.501	**−1.503**	**−0.182**
Realized funct. disp. of sown sp. × time	−0.000	0.003	−0.407	0.405
Full model: marginal *R* ^2^ = .57; conditional *R* ^2^ = .57				

*Note*: In a field experiment, we compared plant communities sown with contrasting seed assemblages and how they affect species establishing spontaneously. We present the results of full averaged models obtained after multimodel inference. Effects whose 95%‐confidence intervals (CI) do not encompass zero are in bold.

## 4 DISCUSSION

We investigated the effects of the initial diversity of flower strip seed assemblages on the realized species richness and functional dispersion of plant traits in a 4‐year field experiment. We designed and compared the dynamics of eight plant assemblages differing in terms of species richness and functional diversity (measured here through functional dispersion). The good agreement between the expected and realized functional dispersion (except in 2014 due to annual weeds) contrasted with previous findings (Hatt et al., 2017). This may reflect seeds being sown in equal numbers rather than equal masses per species, even though we did not take into account possible difference in germination rates. Most (82%) of studies on such semi‐natural habitats have lasted no more than 2 years (Holland et al., 2017). Our 4‐year study made it possible to capture the mid‐term successional dynamics of the assemblages and their consequences for functional composition and diversity. In general, we found strong effects of initial species richness on the functional dynamics of the assemblages, particularly in terms of functional dispersion, probably due to the stabilizing effects of the redundancy generated by higher species richness when functional dispersion remained high. Each of the four types of assemblages was composed of two sets of distinct species, comprising different herbaceous and dicotyledonous species, providing support for the robustness and for the generalizability of our results to other types of herbaceous plant communities.

### 4.1 How do the initial species richness and level of functional dispersion of different plant assemblages affect the multi‐year dynamics of realized functional dispersion?

As expected (De Cauwer et al., 2005; Fritch et al., 2011), we observed a temporal decrease of species richness along the succession in all assemblages. However, this did not modify their functional dispersion in a similar way. In our assemblages, initial richness was manipulated so as to keep functional dispersion constant, such that a gradient of functional redundancy was established. Thus, all assemblages progressively lost species over the 4‐year period, but those with a higher initial species richness and a high functional dispersion (HFHS) remained more functionally diverse over the 4‐year period. These assemblages corresponded to those with the highest degree of functional redundancy at sowing (Table 2). As a result, realized plant functional dispersion remained higher over the whole course of the experiment in species‐rich assemblages than in species‐poor assemblages, confirming our first hypothesis. A possible explanation for this is that functional redundancy would have enabled the compensation for the loss of species by functionally equivalent species (de Bello et al., 2021), attenuating the temporal decrease of functional dispersion and explaining the maintenance of higher levels of functional dispersion for higher species richness. At ecosystem scale, this can have stabilizing effects on ecosystem processes, like conservation biological control (Feit et al., 2019) or pollination (Burkle et al., 2020).

In addition, fewer species were lost over time for higher levels of sown functional dispersion. Similarly, while evenness was initially quite high for all eight assemblages, it declined less over time in situations in which functional dispersion was high. This suggests that there is a higher probability of particular species becoming dominant in assemblages with low functional dispersion, with abundances remaining more evenly distributed in functionally‐rich assemblages. These temporal dynamics of species richness and evenness may have reinforced the compensation effect of functional redundancy, which may explain an overall stabilizing effect on the functional dispersion of the plant assemblages.

In previous studies (Fukami et al., 2005; Roscher et al., 2014), experimental communities initially sown with different plant diversities and species compositions displayed a convergence of species richness and functional composition over time. In this study with the same type of grassland species, it was the case only for assemblages with low or intermediate species richness (9 or 14 species). HFHS assemblages, which were sown with the highest levels of species (29 species) and functional dispersion, remained remarkably separate from the other assemblages and conserved their great dispersion through time. This unexpected result may reflect our exploration of a wider range of species richness (from 9 to 29) than in earlier studies (e.g., 1–16 in the Jena experiment, Roscher et al., 2014). This finding has important implications for restoration projects or for the creation of functionally diversified habitats in agroecosystems, such as birds which respond positively to forb‐rich vegetation (Schmidt et al., 2022). Sowing a sufficiently large number of functionally redundant is necessary to target functionally diverse plant communities.

### 4.2 Can regeneration and competition strategies explain the dynamics in the different assemblages?

As expected, species with early successional traits (i.e., annual or biennial life cycles) were particularly dominant in the first year after sowing, but were subsequently excluded by species with a perennial life cycle and vegetative reproduction (Roscher et al., 2015). This reflects the advantage of species with fast resource acquisition at the beginning of the colonization and the growing importance of more conservative strategies as succession advances (Garnier et al., 2016).

We found no clear temporal dynamics of the community weighted mean of specific leaf area (SLA) across assemblages. The gradient for this trait was very narrow in the studied assemblages, in comparison with the range observed in grasslands or in secondary successions (Garnier et al., 2016). This could be due to the fact that we have sown mostly perennial species, which would have accelerated the succession toward communities with low SLA values. The dynamics of leaf dry matter content (LDMC) were more contrasted. Species with low LDMC, such as *Trifolium pratense* L., *Geum urbanum* L., or *Leucanthemum vulgare* Lam. Were gradually excluded during the 4‐year period of succession. The community‐weighted mean leaf dry matter content of the plants increased over time, consistent with a shift toward species with a strategy of conservation of growth‐related resources. A high LDMC is characteristic of the competitive species commonly found in semi‐natural herbaceous habitats managed by mowing (Cordeau et al., 2017).

Furthermore, the rate of increase in LDMC at community level was inversely related to initial species richness. The assemblages containing the fewest species were the most likely to become dominated by species with high LDMC values, such as *Coronilla varia* L., *Heracleum sphondylium* L., or *Achillea millefolium* L. This finding gave support to our second hypothesis that the temporal dynamics of the communities were driven by competitive exclusion, the strength of which depended on the number of species sown.

### 4.3 Are functionally diversified communities more resistant to invasions by spontaneously arriving species?

Given the role of competitive interactions in community assembly, we expected the diversified assemblages to be more resistant to spontaneous invasion by other species (Galland et al., 2019). Van der Putten et al. (2000) showed that several species‐rich mixtures were more effective at reducing the number of spontaneous species than the average of low‐diversity mixtures. Here, the number of spontaneously arriving species decreased with the functional dispersion of sown species. Consistent with our hypotheses, the arrival of additional species may have been limited by a higher degree of niche occupancy in the functionally diversified communities. For instance, plants with diversified phenologies could limit the establishment of seedlings through a plant cover being developed all year long. This probably contributed to greater resistance to invasion (Carboni et al., 2016; Gallien & Carboni, 2017).

Consistently with our expectations (Hooper & Dukes, 2010; Weisser et al., 2017), the realized functional diversity of the sown species reduced significantly the proportion of the ground covered by spontaneously arriving species. In addition of being less prone to the arrival of new species, the highly diversified assemblages seemed to be more competitive than less diversified assemblages, despite having a lower LDMC. Management options, such as mowing dates and frequencies, are also efficient long‐term levels for managing dominant species, limiting the rate of loss of sown species and limiting the expansion of undesirable spontaneously arriving species (Kirmer et al., 2018).

## 5 CONCLUSIONS

The initial species richness and initial functional dispersion of flower strip seed assemblages were key determinants of multi‐year dynamics of the realized functional dispersion. The assemblages that were initially more diverse maintained their higher levels of functional diversity for several reasons. First, species loss over time was lower for assemblages with a high sown functional dispersion, in which species evenness was maintained at higher levels. Second, species‐rich assemblages had a higher degree of functional redundancy, which may explain why the functional dispersion remained higher over time in conditions of high sown species richness. The realized functional dispersion of the sown species also limited the establishment of spontaneous species (higher degree of niche occupancy). In the assemblages with a low initial species richness, the realized species and functional diversities declined more rapidly, possibly due to stronger competition and the exclusion of poorly competitive species.

This study provides useful insight into the creation and restoration of multifunctional and resilient plant communities supporting higher trophic levels. Such objectives can be achieved by sowing assemblages with a high initial level of species and functional diversity, guaranteeing greater perenniality of the realized communities.

## AUTHOR CONTRIBUTIONS

**Antoine Gardarin:** Conceptualization (lead); data curation (lead); formal analysis (lead); funding acquisition (lead); investigation (lead); methodology (equal); writing – original draft (lead); writing – review and editing (lead). **Muriel Valantin‐Morison:** Conceptualization (supporting); methodology (supporting); supervision (supporting); writing – review and editing (supporting).

## FUNDING INFORMATION

This work was supported by AgroParisTech, INRAE, the “Pour et sur le plan Écophyto 2018” research program, and the “Agence Nationale de la Recherche” (ANR‐12‐AGRO‐0006).

## CONFLICT OF INTEREST

The authors have no competing interests to declare.

## Supporting information


Supporting Information

## Data Availability

The datasets generated during and analyzed during the current study are available in the Zenodo repository: https://doi.org/10.5281/zenodo.4196717.
