# Supplementary material for: Initial assemblage characteristics determine the functional dynamics of flower‐strip plant communities
Source: Ecol Evol. 2022 Oct 18;12(10):e9435. doi: 10.1002/ece3.9435 (PMC9579737; doi:10.1002/ece3.9435)
Supplement: Supplementary file 1 — Supporting Information [file ECE3-12-e9435-s001.pdf]

Initial assemblage characteristics determine the functional dynamics of flower-strip plant communities

Supporting information

Antoine Gardarin and Muriel Valantin-Morison

Supplementary methods. Design of species assemblages with contrasting species and functional diversities

Based on the BiolFlor trait database (Kühn et al., 2004), we classified plant species into 12 functional groups (Table S1) according to their traits. As we were interested in mixtures enhancing conservation biological control, we focused on traits involved in plant-arthropod interactions, easily accessible in databases: (1) flower resources, i.e. floral and extrafloral nectar or pollen, (2) accessibility of the resource, depending on flower shape, (3) availability of the resource, i.e. the duration of the flowering period and (4) height at flowering. These traits were only used for creating the mixtures, but a larger range of traits has been used for the analyses presented in this study (see Material and Methods).

We constructed the high functional diversity - medium species richness diversity (HFMS) assemblages by choosing species from each of the 12 functional groups identified in Supporting information Table S1. The low functional diversity - medium species richness (LFMS) assemblages were obtained by reducing the number of functional groups to seven and increasing the number of species per group, so as to keep species richness constant. In the HFLS assemblages, we retained only the species of the HFMS assemblages belonging the most highly contrasting functional groups, to obtain the highest diversity possible. Although the HFLS assemblages contain less functional groups (6) than the LFMS ones (7), the functional groups in the former are more contrasted than in the latter, with very early flowering species (functional group 9), short plant species (functional groups 8 and 9) and with species producing extrafloral nectar (functional group 10). In the HFHS assemblages, we increased species richness and kept functional diversity as constant as possible by adding extra species with trait combinations closely resembling those already present in the HFMS assemblages.

We then added the same three tussock grass species to each assemblage, in similar quantities (Table S2).

Table S1. List of the plant functional groups included in the four types of assemblages (LF and HF mean a low or high functional diversity, LS, MS or HS mean a low, medium or high species richness respectively). Traits values were taken from the BiolFlor (Kühn et al., 2004) and LEDA (Kleyer et al., 2008) trait databases.

Functional group	Flower class after Müller (1881)	Flower type after Kugler (1970)	Month of flowering onset	Month of flowering end	Presence of nectar	Amount of pollen	Extra-floral nectar	Plant height	LFMS	HFLS	HFMS	HFHS
1	flowers with open nectar	disk flowers with nectar open	6-7	9	present	present	none	medium to tall	x	x	x	x
2	flowers with partly hidden nectar	disk flowers with nectar ± hidden nectaries at base of stamens	4	6	present	present	none	medium to tall	x	x	x	x
3	flower associations with totally hidden nectar	flower heads, Asteraceae, ray and disk flowers	6	9-10	present	present	none	medium to tall	x	x	x	x
4	flowers with open or partl hidden nectar	disk flowers with nectar open or nectar ± hidden in centre of flower	4-5	6-7	present	present	none	medium to tall	x		x	x
5	flowers with totally hidden nectar	stalk disc flowers, stamina and pistil within tube	5	7	present	present	none	medium to tall	x			x
6	flower associations with totally hidden nectar	flower heads, Asteraceae or non-Asteraceae	7	9-10	present	present	none	tall	x		x	x
7	hymenoptera flowers	flag blossom, Fabaceae type	6	7 to 9	present	present	none	medium to tall	x			x
8	hymenoptera flowers	flag blossom, Fabaceae type or true lip flowers	4-5	6 to 9	present	present	none	low		x	x	x
9	flowers with totally hidden nectar	several types	1 to 3	5	present	present	none	low		x	x	x
10	flower associations with totally hidden nectar or hymenoptera flowers	flower head or flag blossom	5 to 6	7	present	present	present	medium to tall		x	x	x
11	pollen flowers	pollen flowers	5 to 7	8 to 9	none	plenty	none	medium to tall			x	x
12	flowers with partly or totally hidden nectar	disk flowers with nectar ± hidden	5-6	8 to 9	present	present	none	medium to tall				x

Müller H (1881) Alpenblumen, ihre Befruchtung durch Insekten und ihre Anpassungen an dieselben. W. Engelmann, Leipzig
Kleyer M, Bekker RM, Knevel IC, Bakker JP, Thompson K, Sonnenschein M, Poschlod P, Van Groenendael JM, *et al.* (2008) The LEDA Traitbase: a database of life-history traits of the Northwest European flora. Journal of Ecology 96 (6):1266-1274. doi:10.1111/j.1365-2745.2008.01430.x
Kugler H (1970) Blütenökologie. 2nd edn. Gustav Fischer, Jena,
Kühn, I., Durka, W., Klotz, S., 2004. BiolFlor - a new plant-trait database as a tool for plant invasion ecology. Diversity and Distributions 10, 363-365.

Table S2. Origin and perenniality of the sown plant species. Seeds produced by *Ecosem* were produced locally (northern France).

Species	Seed provider	Perenniality	Species	Seed provider	Perenniality
Achillea millefolium L.	Ecosem	Perennial	*Hypochaeris radicata* L.	Herbiseed	Perennial
Ajuga reptans L.	Phytosem	Perennial	*Jacobaea vulgaris* L.	Phytosem	Perennial
Alliaria petiolata Cavara&Grande	Herbiseed	Biennial	*Knautia arvensis* (L.) Coult.	Ecosem	Perennial
Anthriscus sylvestris (L.) Hoffm.	Ecosem	Perennial	*Lamium album* L.	Herbiseed	Perennial
Arctium minus (Hill) Bernh.	Herbiseed	Perennial	*Leucanthemum vulgare* Lam.	Ecosem	Perennial
Arrhenatherum elatius (L.) P.Beauv.	Phytosem	Perennial	*Lotus corniculatus* L.	Ecosem	Perennial
Barbarea vulgaris R. Br.	Ecosem	Biennial	*Malva sylvestris* L.	Ecosem	Perennial
Bellis perennis L.	Ecosem	Perennial	*Medicago lupulina* L.	Les semences du Puy	Perennial
Capsella bursa-pastoris (L.) Med.	Phytosem	Annual	*Medicago sativa* L.	Ecosem	Perennial
Carum carvi L.	Herbiseed	Perennial	*Melilotus altissimus* Thuill.	Phytosem	Perennial
Centaurea scabiosa L.	Ecosem	Perennial	*Onobrychis viciifolia* Scop.	Herbiseed	Perennial
Cichorium intybus L.	Ecosem	Biennial	*Pastinaca sativa* L.	Ecosem	Biennial
Coronilla varia L.	Ecosem	Perennial	*Plantago lanceolata* L.	Phytosem	Perennial
Cota tinctoria (L.) J.Gay ex Guss.	Phytosem	Biennial	*Potentilla reptans* L.	Herbiseed	Perennial
Cyanus segetum L.	Phytosem	Annual	*Ranunculus repens* L.	Phytosem	Perennial
Cynoglossum officinale L.	Les semences du Puy	Biennial	*Reseda luteola* L.	Ecosem	Perennial
Dactylis glomerata L.	Phytosem	Perennial	*Schedonorus arundinaceus* Schreb.	Phytosem	Perennial
Daucus carota L.	Ecosem	Biennial	*Stellaria media* (L.) Vill.	Phytosem	Annual
Echium vulgare L.	Ecosem	Perennial	*Tanacetum vulgare* L.	Ecosem	Perennial
Euphorbia cyparissias L.	Herbiseed	Perennial	*Taraxacum sect. Ruderalia* Wiggers	Phytosem	Perennial
Foeniculum vulgare Mill.	Phytosem	Perennial	*Trifolium pratense* L.	Phytosem	Perennial
Galium mollugo L.	Ecosem	Perennial	*Trifolium repens* L.	Ecosem	Perennial
Galium odoratum (L.) Scop.	Herbiseed	Perennial	*Verbascum densiflorum* Bertol.	Les semences du Puy	Biennial
Geum urbanum L.	Herbiseed	Perennial	*Verbascum lychnitis* L.	Les semences du Puy	Biennial
Glechoma hederacea L.	Les semences du Puy	Perennial	*Veronica hederifolia* L.	Herbiseed	Annual
Heracleum sphondylium L.	Herbiseed	Perennial	*Veronica persica* Poir.	Les semences du Puy	Annual
Hesperis matronalis L.	Phytosem	Biennial	*Vicia sativa* L.	Les semences du Puy	Annual
Hypericum perforatum L.	Arbiotech	Perennial			

Table S3. Effects and 95% confidence intervals of the characteristics of the initial plant assemblages on the temporal dynamics of the composition and diversity of the resulting plant communities over a period of four years. In complement to the results of Table 3, we analysed separately the gradients of initial functional dispersion and species richness. The effect of initial functional dispersion was analysed on a subset of medium species richness assemblages (left panel). The effect of initial richness was analysed on the subset of high functional dispersion assemblages (right panel). Effects whose confidence interval do not encompass zero are in bold. When there was only one best model selected after multimodel inference, we present the results of the analysis of variance of this best model.

Only medium species richness assemblages (LFMS and HFMS)

Response variable: functional dispersion of the whole plant community

		Full averaged model	
**Explanatory fixed variables**	**Factor levels**	**Effect**	**CI**
Intercept		0.229	0.206, 0,251
Time		**-0.022**	**-0.029, -0.015**
Initial funct. disp. (ref = low)	high	**0.032**	**0.007, 0.057**
Initial funct. disp × time (ref = low)	high × time	-0.005	-0.017, 0.006

Marginal R^2 = 0.46; conditional R^2 = 0.53

Response variable: standardized effect size of functional dispersion of the whole plant community (calculated with a null model)

		Analysis of variance	
**Explanatory fixed variables**	**Factor levels**	**Effect**	**CI**
Intercept		-0.686	-1.440, 0,067
Time		**-0.309**	**-0.575, -0.042**
Initial funct. dispersion (ref = low)	high	**3.078**	**2.012, 4.144**
Initial funct. disp × time (ref = low)	high × time	**-0.821**	**-1.197, -0.444**

Marginal R^2 = 0.53; conditional R^2 = 0.57

Response variable: species richness of the whole plant community

		Full averaged model	
**Explanatory fixed variables**	**Factor levels**	**Effect**	**CI**
Intercept		3.500	3.617, 3.681
Time		**-0.160**	**-0.223, -0.097**
Initial funct. dispersion (ref = low)	high	-0.097	-0.400, 0.097
Initial funct. disp × time (ref = low)	high × time	**0.042**	**0.006, 0.161**

Marginal R^2 = 0.34; conditional R^2 = 0.49

Only high functional dispersion assemblages (HFLS, HFMS and HFHS)

Response variable: functional dispersion of the whole plant community

		Analysis of variance	
**Explanatory fixed variables**	**Factor levels**	**Effect**	**CI**
Intercept		0.245	0.221, 0.270
Time		**-0.036**	**-0.044, -0.028**
Initial species richness (ref = low)	medium	0.019	-0.014, 0.052
	high	0.013	-0.020, 0.047
Initial sp. richness × time (ref = low)	medium×time	**0.011**	**0.001, 0.035**
	high × time	**0.023**	**0.012, 0.035**

Marginal R^2 = 0.62; conditional R^2 = 0.70

Response variable: standardized effect size of functional dispersion of the whole plant community (calculated with a null model)

		Analysis of variance	
**Explanatory fixed variables**	**Factor levels**	**Effect**	**CI**
Intercept		2.683	1.826, 3.528
Time		**-1.614**	**-1.909, -1.315**
Initial species richness (ref = low)	medium	-0.267	-1.454, 0.921
	high	-0.715	-1.902, 0.473
Initial sp. richness × time (ref = low)	medium×time	**0.479**	**0.059, 0.899**
	high × time	**0.872**	**0.452, 1.292**

Marginal R^2 = 0.64; conditional R^2 = 0.65

Response variable: species richness of the whole plant community

		Full averaged model	
**Explanatory fixed variables**	**Factor levels**	**Effect**	**CI**
Intercept		3.136	2.999, 3.274
Time		**-0.088**	**-0.124, -0.051**
Initial species richness (ref = low)	medium	**0.194**	**0.072, 0.315**
	high	**0.477**	**0.359, 0.596**
Initial sp. richness × time (ref = low)	medium×time	-0.000	-0.083, 0.079
	high × time	0.003	-0.057, 0.095

Marginal R^2 = 0.50; conditional R^2 = 0.59

Table S3. (continued)

Only medium species richness assemblages (LFMS and HFMS)

Response variable: Simpson's species evenness of the whole plant community *

		Full averaged model	
**Explanatory fixed variables**	**Factor levels**	**Effect**	**CI**
Intercept		0.866	0.769, 0.963
Time		-0.007	-0.061, 0.048
Initial funct. dispersion (ref = low)	high	**-0.117**	**-0.224, -0.010**
Initial funct. disp × time (ref = low)	high × time	0.044	-0.006, 0.093

Marginal R^2 = 0.14; conditional R^2 = 0.35

Response variable: CWM of LDMC of the whole plant community

		Full averaged model	
**Explanatory fixed variables**	**Factor levels**	**Effect**	**CI**
Intercept		178.370	165.223, 191.517
Time		**12.514**	**8.051, 16.977**
Initial funct. dispersion (ref = low)	high	-8.626	-30.175, 12.924
Initial funct. disp × time (ref = low)	high × time	5.733	-0.216, 11,683

Marginal R^2 = 0.42; conditional R^2 = 0.60

Only high functional dispersion assemblages (HFLS, HFMS and HFHS)

Response variable: Simpson's species evenness of the whole plant community *

		Full averaged model	
**Explanatory fixed variables**	**Factor levels**	**Effect**	**CI**
Intercept		0.711	0.616, 0.805
Time		**0.023**	**0.004, 0.056**
Initial species richness (ref = low)	medium	**0.096**	**0.030,0.222**
	high	**0.110**	**0.010, 0.210**
Initial sp. richness × time (ref = low)	medium×time	**-0.035**	**-0.076, -0.013**
	high × time	-0.013	-0.053, 0.020

Marginal R^2 = 0.16; conditional R^2 = 0.23

Response variable: CWM of LDMC of the whole plant community

		Analysis of variance	
**Explanatory fixed variables**	**Factor levels**	**Effect**	**CI**
Intercept		173.545	160,406, 186,660
Time		**20.193**	**15.751, 24.636**
Initial species richness (ref = low)	medium	-3.916	-22.383, 14.451
	high	2.245	-16.183, 20.644
Initial sp. richness × time (ref = low)	medium×time	**-3.779**	**-8.053, -0.505**
	high × time	**-9.497**	**-15.774, -3.216**

Marginal R^2 = 0.58; conditional R^2 = 0.63

* The three lowest values were outliers and were removed to reach normality.

Table S4. Effects of the characteristics of the initial plant assemblages on the temporal dynamics of the standardized functional dispersion (standardized difference between the observed values and values from a null model). As there was only one best model selected after multimodel inference, we present the results of the analysis of variance of this best model. P-values were computed from Wald χ^2 tests. P-values lower than 0.05 are written in bold. In contrast to results of Table 3, time was modeled as a categorical « year » variable to account for the non-linear temporal dynamics of the response variable.

**Response variable: standardized functional dispersion**						
**Explanatory fixed variables**	χ^2	**df**	**P (>χ^2)**	**Factor levels**	**Effect ± SE**	**Significant differences after all pair-wise comparisons**
Year (ref = 2014)	410.911	3	**< 10^{-4}**	2015	-1.262 ± 0.458	2015 < 2014
				2016	-0.849 ± 0.372	2016 < 2014
				2017	-0.520 ± 0.373	2017 < 2014
Initial species richness (ref = low)	36.822	2	**< 10^{-4}**	medium	0.010 ± 0.217	medium > low
				high	0.103 ± 0.217	high > low
Initial sp. richness × year (ref = low, 2014)	21.698	6	**$1.374 \cdot 10^{-3}$**	2015, medium	0.709 ± 0.374	
				2015, high	0.277 ± 0.374	
				2016, medium	0.367 ± 0.304	2016: medium, high > low
				2016, high	0.912 ± 0.304	
				2017, medium	0.441 ± 0.304	2017: high > low, medium
				2017, high	1.106 ± 0.304	
Initial funct. dispersion (ref = low)	31.768	1	**< 10^{-4}**	high	2.064 ± 0.217	high > low
Initial funct. disp × year (ref = low, 2014)	64.442	3	**< 10^{-4}**	2015, high	-1.386 ± 0.374	2014: high > low
				2016, high	-1.936 ± 0.304	
				2017, high	-2.257 ± 0.304	
Marginal R^2 = 0.78; conditional R^2 = 0.79						

Table S5. Effects of the characteristics of the initial plant assemblages on the temporal dynamics of the community weighted mean of specific leaf area. Effects whose confidence interval do not encompass zero are in bold.

**Response variable: community-weighted mean of SLA for the whole plant community**				
**Explanatory fixed variables**	**Factor levels**	**Effect**	**z value**	**CI**
Intercept		23.465	26.274	21.715, 25.216
Time		**0.747**	1.981	**0.008, 1.485**
Initial species richness (ref = low)	medium	-0.108	0.175	-1.716, 1.360
	high	-0.331	0.647	-1.647, 0.554
Initial sp. richness × time (ref = low)	medium×time	-0.023	0.173	-0.987, 0.007
	high × time	-0.124	0.499	-0.588, 0.406
Initial funct. dispersion (ref = low)	high	0.809	0.968	-0.591, 2.615
Initial funct. disp × time (ref = low)	high × time	-0.420	1.197	-1.116, 0.066
Marginal R^2 = 0.14; conditional R^2 = 0.24				

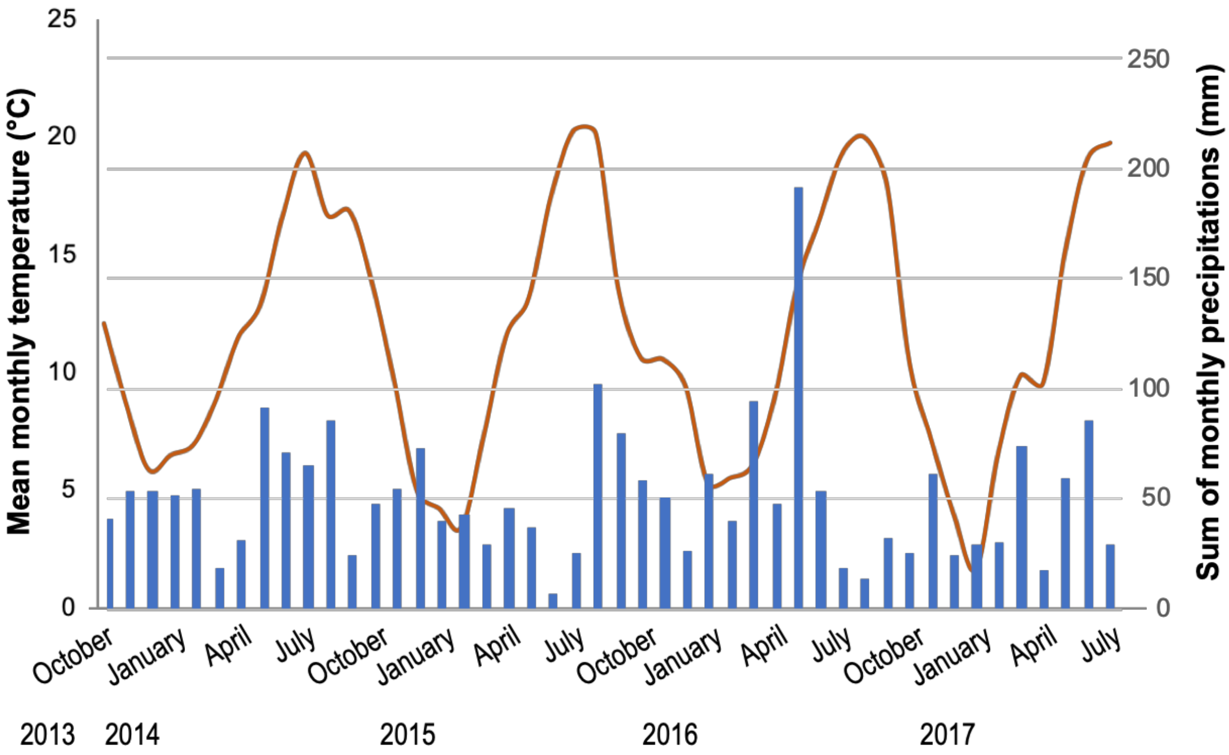

Figure S1. Mean monthly temperature (orange curve, left axis) and monthly precipitations (blue bars, right axis) during the four-year experiment.

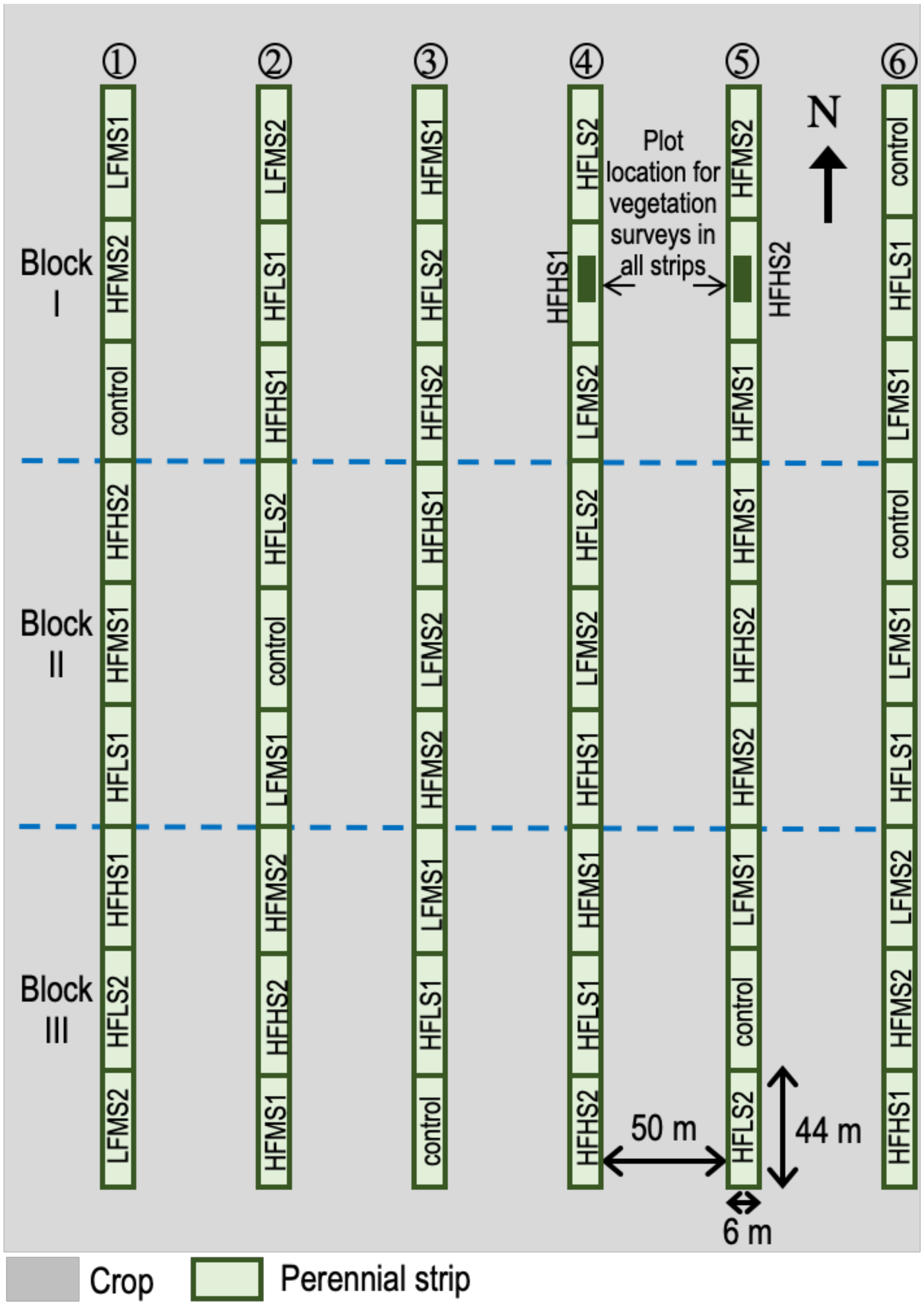

Figure S2. Design of the experimental field. The control plots were not used in this study. The eight plant assemblages have a low or high functional dispersion (LF or HF), a low, medium or high species richness (LS, MS or HS) and are composed of species from two different lists (Table 2).

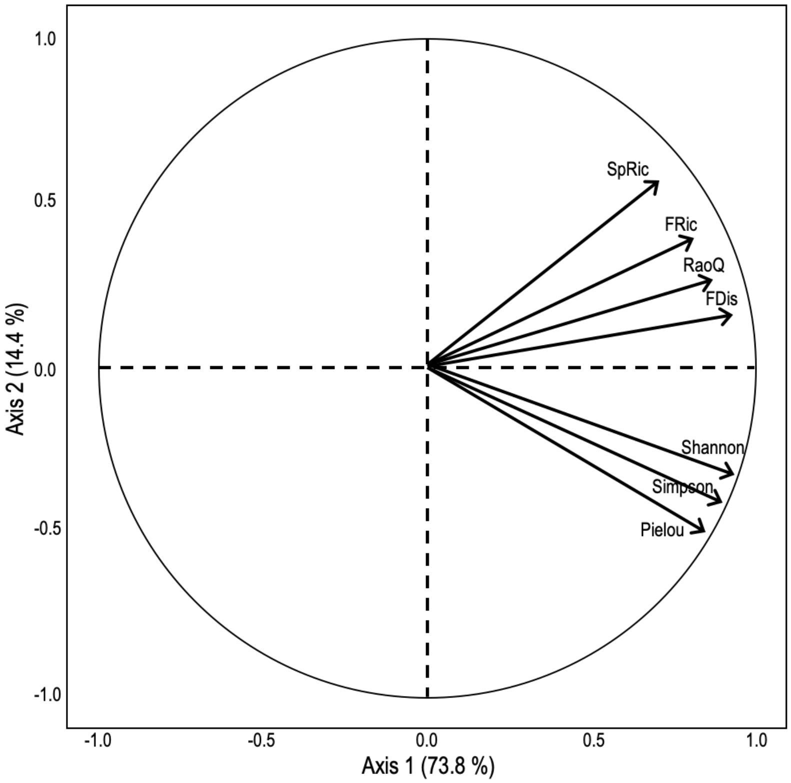

Figure S3. Principal component analysis performed on the observed plant communities during the four-year experiment described by several metrics of taxonomic and functional diversity: species richness (SpRic), Shannon's species diversity (Shannon), Simpson's species evenness (Simpson), Pielou's species evenness index (Pielou), functional dispersion (FDis), functional richness (FRic), and Rao quadratic entropy (RaoQ).

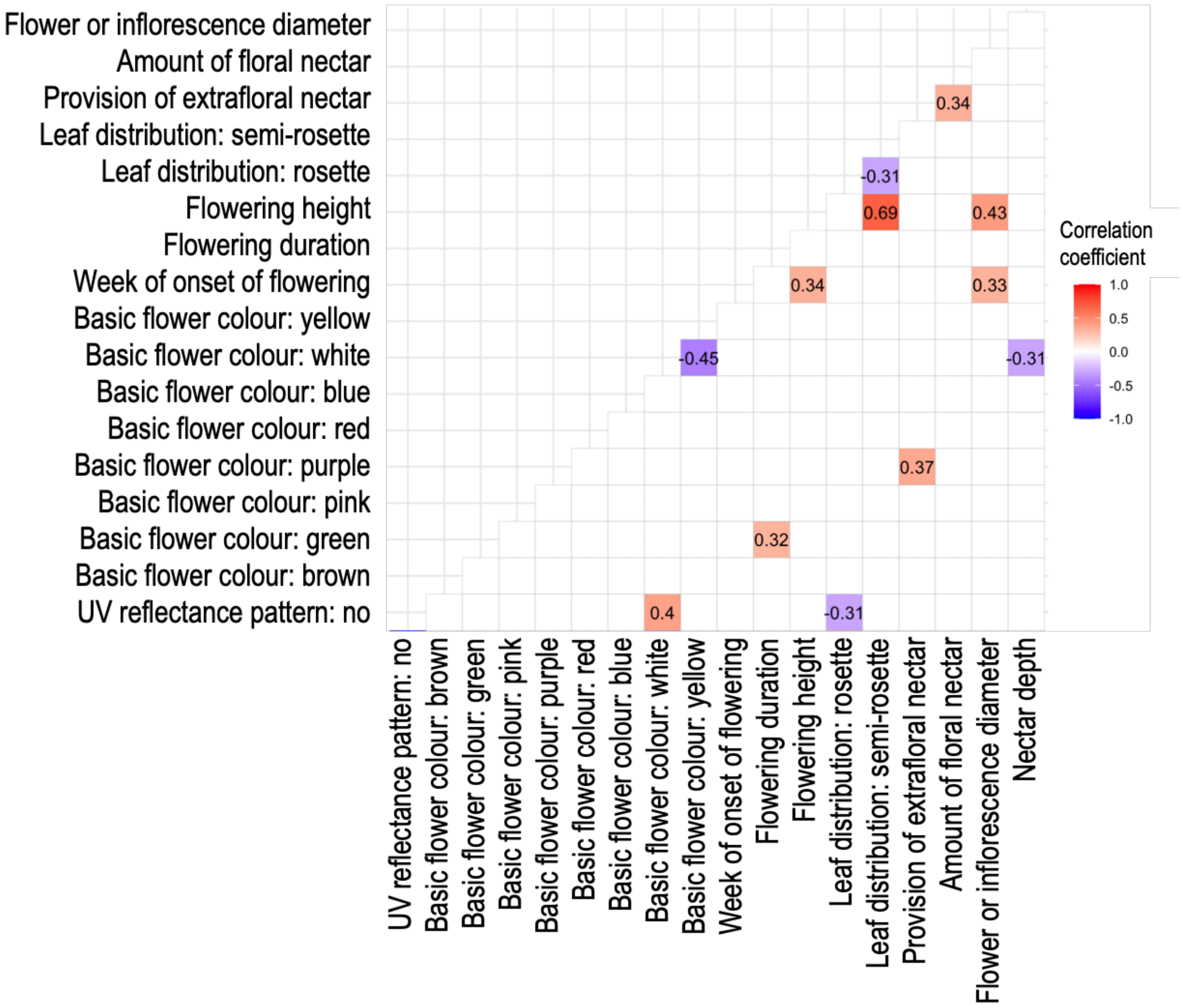

Figure S4. Correlation plot (drawn with the library ggcorrplot under R) between the traits involved in plant-arthropod interactions. Only correlation coefficients significant at $P < 0.05$ are shown. Factor levels were considered as separate variables for categorical traits. We did not interpret correlations between the trait attributes within a single trait (e.g. between colors) which are mutually exclusive.

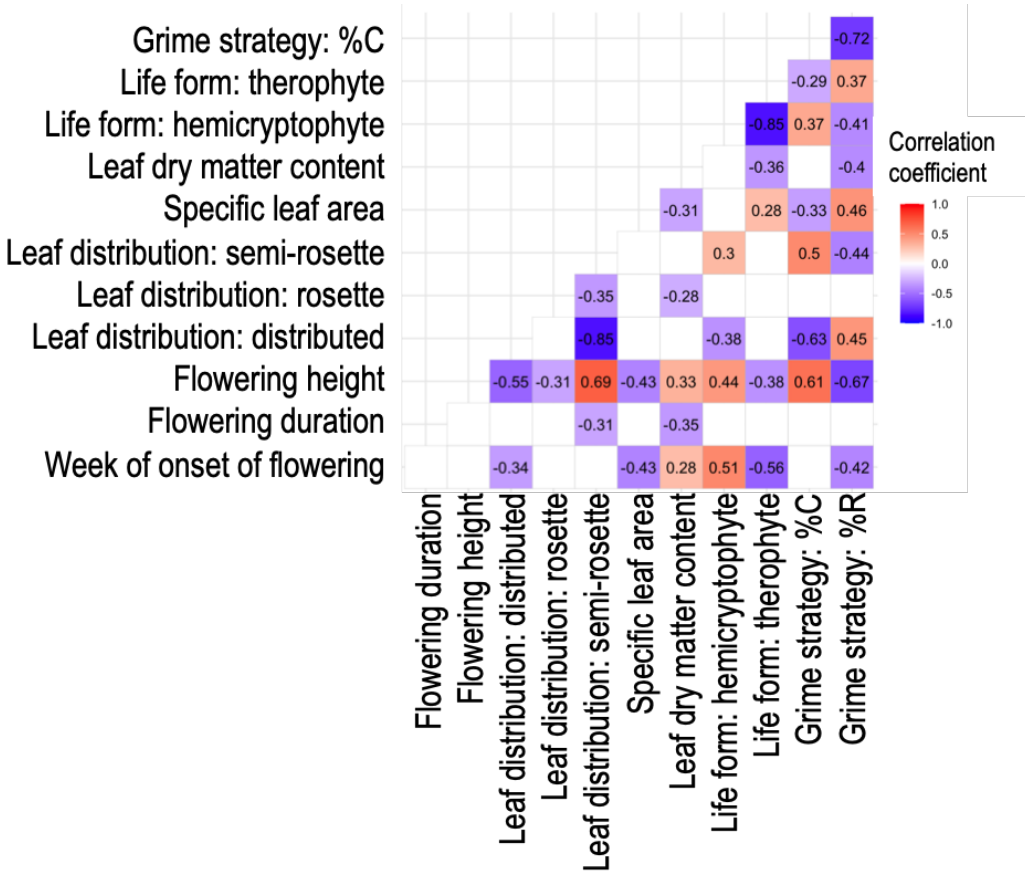

Figure S5. Correlation plot (drawn with the library ggcorrplot under R) between the traits involved in plant-plant interactions. Only correlation coefficients significant at P < 0.05 are shown. Factor levels were considered as separate variables for categorical traits. We did not interpret correlations between the trait attributes within a single trait (e.g. between life forms or Grime strategies) which are mutually exclusive.
